# Systematic Determination of Herpesvirus in Free-Ranging Cetaceans Stranded in the Western Mediterranean: Tissue Tropism and Associated Lesions

**DOI:** 10.3390/v13112180

**Published:** 2021-10-28

**Authors:** Ignacio Vargas-Castro, Mar Melero, José Luis Crespo-Picazo, María de los Ángeles Jiménez, Eva Sierra, Consuelo Rubio-Guerri, Manuel Arbelo, Antonio Fernández, Daniel García-Párraga, José Manuel Sánchez-Vizcaíno

**Affiliations:** 1 VISAVET Health Surveillance Centre and Animal Health Department, Veterinary School, Complutense University of Madrid, 28040 Madrid, Spain; mar.melero@correo.gob.es (M.M.); consuelo.rubio@uchceu.es (C.R.-G.); jmvizcaino@ucm.es (J.M.S.-V.); 2 Division of External Health, Government Delegation in the Community of Madrid, Ministry of Territorial Policy, 28071 Madrid, Spain; 3 Research Department, Fundación Oceanogràfic de la Comunitat Valenciana, 46013 Valencia, Spain; jlcrespo@oceanografic.org (J.L.C.-P.); dgarcia@oceanografic.org (D.G.-P.); 4 Department of Animal Medicine and Surgery, Veterinary Faculty, Complutense University of Madrid, 28040 Madrid, Spain; mariadji@ucm.es; 5 Division of Veterinary Histology and Pathology, Institute for Animal Health, Veterinary School, University of Las Palmas de Gran Canaria, 35416 Canary Islands, Spain; eva.sierra@ulpgc.es (E.S.); manuel.arbelo@ulpgc.es (M.A.); antonio.fernandez@ulpgc.es (A.F.); 6 Department of Pharmacy, Facultad de CC de la Salud, UCH-CEU University, 46113 Valencia, Spain

**Keywords:** dolphin, herpesvirus, latency, marine mammal, coinfection, morbillivirus, mRNA, alphaherpesvirus, gammaherpesvirus, CNS

## Abstract

The monitoring of herpesvirus infection provides useful information when assessing marine mammals’ health. This paper shows the prevalence of herpesvirus infection (80.85%) in 47 cetaceans stranded on the coast of the Valencian Community, Spain. Of the 966 tissues evaluated, 121 tested positive when employing nested-PCR (12.53%). The largest proportion of herpesvirus-positive tissue samples was in the reproductive system, nervous system, and tegument. Herpesvirus was more prevalent in females, juveniles, and calves. More than half the DNA PCR positive tissues contained herpesvirus RNA, indicating the presence of actively replicating virus. This RNA was most frequently found in neonates. Fourteen unique sequences were identified. Most amplified sequences belonged to the *Gammaherpesvirinae* subfamily, but a greater variation was found in *Alphaherpesvirinae* sequences. This is the first report of systematic herpesvirus DNA and RNA determination in free-ranging cetaceans. Nine (19.14%) were infected with cetacean morbillivirus and all of them (100%) were coinfected with herpesvirus. Lesions similar to those caused by herpesvirus in other species were observed, mainly in the skin, upper digestive tract, genitalia, and central nervous system. Other lesions were also attributable to concomitant etiologies or were nonspecific. It is necessary to investigate the possible role of herpesvirus infection in those cases.

## 1. Introduction

Marine mammals such as cetaceans have been described as good sentinels of marine ecosystem health because they have long lifespans, reside along coasts for long periods, occupy a high trophic level, and have blubber stores that can serve as depots for anthropogenic chemicals and toxins [1]. Moreover, these animals are vulnerable to infectious diseases, some of which have implications for human public health and others of which may serve as indicators of environmental distress syndrome [1]. For example, several studies have reported herpesvirus (HV) infection in cetaceans [2,3,4,5,6,7,8,9,10,11,12,13,14,15,16,17,18,19,20,21,22,23], with prevalences of up to 62.5% [24] or 78.57% [21]. HV can establish latent infections [25,26], and stress and immunosuppression can then cause the virus to revert to an actively replicating state [27]. Monitoring HV infection and determining whether the virus is latent or actively replicating in certain tissues can, therefore, provide useful information with which to assess marine mammals’ health.

The *Herpesviridae* family, which comprises the subfamilies *Alphaherpesvirinae*, *Betaherpesvirinae,* and *Gammaherpesvirinae*, can infect a wide range of mammalian species, birds, reptiles, fish, frogs, and bivalves [28]. All HV detected in marine mammals to date belong to the *Alpha*- or *Gammaherpesvirinae* subfamilies [29].

With regard to cetaceans, HV was first detected in the dermatitis-related skin lesions of a beluga whale (*Delphinapterus leucas*) [2,3], and HV-like particles were later detected in the skin lesions of a dusky dolphin (*Lagenorhynchus obscurus*) [5] and in a harbor porpoise (*Phocoena phocoena*) with encephalitis [4]. Subsequent studies have identified *Alphaherpesvirinae* in numerous disease contexts: interstitial nephritis in a Blainville’s beaked whale (*Mesoplodon densirostris*) [13,30]; non-suppurative encephalitis in a bottlenose dolphin (*Tursiops truncatus*) [10], harbor porpoise [23], and striped dolphin (*Stenella coeruleoalba*) [15]; skin lesions on an Atlantic bottlenose dolphin [7,9], striped dolphin [15], Guiana dolphin (*Sotalia guianensis*), and dwarf sperm whale (*Kogia sima*) [22]; penile proliferative lesions in a beluga whale [16] and a disseminated infection with acute necrotizing lesions in an Atlantic bottlenose dolphin [6]; necrotizing lymphadenitis and splenitis in a Cuvier’s beaked whale (*Ziphius cavirostris*) [12,30]; and severe lymphoid depletion and necrosis in a striped dolphin [14]. This has also been determined on the buffy coat of a bottlenose dolphin, in blowhole exudate from an orca (*Orcinus orca*) [19], and on the skin and in the penile mucosa of a fin whale (*Balaenoptera physalus*) [17]. *Gammaherpesvirinae* has been associated with genital lesions on a dwarf sperm whale, Risso’s dolphin (*Grampus griseus*) [9], bottlenose dolphin [9,11,31], Blainville’s beaked whale [8,9], common dolphin (*Dephinus delphis*) [20], Guiana dolphin [32], harbor porpoise [23], and striped dolphin [18,21]; proliferative dermatitis has been found on a Bolivian river dolphin (*Iniaboliviensis*) [22] and has been amplified from the skin, muscle, and central nervous system (CNS) of a common minke whale (*Balaenoptera acutorostrata*) [17]. However, HV DNA has also been reported in cetaceans in the absence of any lesions [21,23,24,30,33,34]. Moreover, HV can cause immunosuppression in cetaceans [12] and in several other animal species [35,36,37], including humans [38,39].

HV can establish a latent infection, during which it expresses only a small subset of viral genes and no viral particles are produced [25,26]. This possibility of latent infection means that detecting HV DNA in animal tissues does not necessarily indicate active viral replication. Indeed, many cases of HV-related disease have been linked to the reactivation of latent virus rather than to primary infection [40].

Cetaceans are useful as marine sentinels, and detailed and reliable knowledge about the diseases and infectious agents that can affect them is, therefore, required. Great progress has been made regarding the study of HV in these animals in recent years, although much remains to be discovered. One study examining various tissues in striped dolphins found that 5 out of 8 animals (62.5%) tested positive when employing PCR [24]; a study of the seroprevalence of antibodies against HV in beluga whales found that 6 out of 13 animals (46.15%) were positive when employing a serum neutralization test, while 7 out of 12 (58.33%) tested positive in the case of a blocking enzyme-linked immunosorbent assay (ELISA) [41]. Further work is, therefore, required in order to understand how HV prevalence is influenced by specific cetacean species, along with their sex, age, and tissues.

The objective of this study is to provide an insight into the prevalence of HV in free-ranging cetaceans by studying samples obtained from animals stranded on the Mediterranean coast of the Valencian Community, Spain, in terms of viral DNA and RNA detection, tissue tropism, and the relevance of this prevalence in terms of lesions and disease. The samples were analyzed over three consecutive years. Several of these animals were also diagnosed as having cetacean morbillivirus infection (CeMV); CeMV has also been reported to cause immunosuppression [42,43] and to co-occur with HV in cetaceans [14,15,20,24].

## 2. Materials and Methods

### 2.1. Animals

Analyses were carried out on forty-seven cetaceans stranded on the Mediterranean coast of the Valencian Community (Spain) between June 2010 and June 2013: 35 striped dolphins, 8 bottlenose dolphins, 3 Risso’s dolphins, and 1 Cuvier’s beaked whale. Individuals were classified into four age groups (17 adults, 18 juveniles, 4 calves, and 7 neonates) estimated on the basis of the total length of the animal, teeth eruption, the presence of thymus, gonad maturity, and the radiological analysis of the pectoral flipper [44,45,46,47,48]. Age remained undetermined for one individual and sex was undetermined for four individuals owing to the conservation status of these animals.

The Oceanogràfic is part of the Stranding Network, as the result of an agreement involving the Fundación Oceanogràfic; Ciudad de las Artes y las Ciencias; and the Conselleria d’Agricultura, Desenvolupament Rural, Emergència Climàtica i Transició Ecològica. This agreement led the Regional Government (Generalitat Valenciana) to authorize the Oceanogràfic to lead and perform veterinary assistance in cases of stranded cetaceans. This agreement includes participation in the related necropsies (together with the University of Valencia), and allows samples collected from carcasses to be used for research purposes. All samples were collected by the authors (licensed veterinarians) during the necropsy. According to European Parliament and Council legislation 2010/63/UE (22 September 2010) and Spanish Royal Decree 53/2013 (1 February 2013), approval from the corresponding Ethical Committee is not required when post-mortem tissue is collected for research purposes. Five of the 47 animals were euthanized for humanitarian reasons, following the recommended guidelines [49,50]. No animals were specifically euthanized, nor were blood and tissue samples collected from live animals, solely for the purpose of this study, thus approval from the corresponding Ethical Committee was not required.

Necropsies were performed in accordance with the standard procedures of the European Cetacean Society [51]. When transport to a necropsy room was not feasible owing to carcass condition or the size of the animal, samples were collected at the stranding site (in the case of four stranded cetaceans). The state of carcass preservation was classified using the criteria suggested by Geraci and Lounsbury [52].

A total of 966 samples of 48 different tissues and fluids (Table 1) were collected and stored at −80 °C. Tissue samples were homogenized using a Bullet Blender^TM^ (Next Advance, Averill Park, NY, USA). Teeth were separated from the jaw and broken to allow pulp extraction during homogenization, together with the gum and bone remains.

### 2.2. Herpesvirus Detection

Total DNA was extracted using the High Pure Template Preparation Mix according to the manufacturer’s instructions (Roche Diagnostics, Mannheim, Germany).

All samples were subjected to panherpesvirus nested polymerase chain reaction (PCR), targeting the DNA polymerase gene [53]. All positive PCR products were purified using a PCR Purification kit (Qiagen, Germantown, MD, USA) and were subsequently sequenced.

### 2.3. Phylogenetic Analysis

Sequences were aligned using MEGA 5.2 software (Tokyo, Japan) [54]. The accuracy of the alignment and, therefore, its ability to produce reliable phylogenetic trees, was assessed by calculating average amino acid p-distance (1-amino acid identity), in which <0.8 was taken to be the threshold of acceptability [55,56]. Sequences were subjected to phylogenetic analysis, and a phylogenetic tree based on amino acid sequences was constructed using the maximum likelihood (ML) method and the Jones–Taylor–Thornton model with gamma-distributed rates with invariant sites (JTT+G+I) in MEGA 5.2 [54]. A bootstrap consensus tree was inferred from 500 replications.

### 2.4. Herpesvirus RNA Detection to Assess Active Replication

In addition to testing for the presence of HV DNA, samples were also tested for viral messenger RNA (mRNA) in order to detect actively replicating virus, as has occurred previously in HV studies carried out on marine mammals [57]. Total RNA was extracted from all positive samples using the NucleoSpin RNA II Kit (Macherey-Nagel, Düren, Germany), and was then reverse-transcribed using the Affinity Script QPCR cDNA Synthesis kit according to the manufacturer’s instructions (Agilent Technologies, Santa Clara, CA, USA). The resulting complementary DNA then served as a template in the same panherpesvirus nested PCR described above. In order to ensure that the RNA extracted from the samples was of sufficient quality to provide reliable results, the extracts were subjected to reverse transcription-PCR (RT-PCR) so as to amplify the housekeeping gene encoding glyceraldehyde-3-phosphate dehydrogenase (GAPDH), as previously described [58]. Only samples for which the GAPDH RNA was detected were included in the viral latency assays.

Furthermore, in order to ensure that the positive results regarding RNA determination were not attributable to interference resulting from the possible presence of DNA remains, PCRs were also performed in parallel using the RNA extracted without the presence of reverse transcriptase, thus ruling out false positive results.

### 2.5. Herpesvirus and Morbillivirus Coinfection

In order to detect coinfection with HV and CeMV, all animals were additionally tested for CeMV using a real-time RT-PCR [59] on samples of skin, brain, lung, kidney, and prescapular and pulmonar lymph nodes.

### 2.6. Histopathological Study

Tissue samples from the mammary gland, genital mucosa, uterus, ovary, testicle, meninges, brain, cerebellum, spinal cord, skin, blubber, thyroid, adrenal gland, heart, kidney, urinary bladder, esophagus, liver, pancreas, lung, spleen, lymph nodes (pre-escapular, pulmonar, and mesenteric), pharyngeal tonsils, thymus, and skeletal muscle were collected when the carcass-preservation conditions were adequate, fixed in 10% buffered formalin, trimmed and processed according to routine laboratory procedures for histologic examination with hematoxylin-eosin stains. Only those samples that tested positive for HV in the molecular diagnosis were taken into account for the histopathological study.

## 3. Results

### 3.1. Herpesvirus Detection

Of the 47 cetaceans tested, 38 (80.85%) were positive for HV in at least one tissue. Of the 966 tissue samples tested, 121 (12.53%) were positive (Table 1).

**Figure 1:**
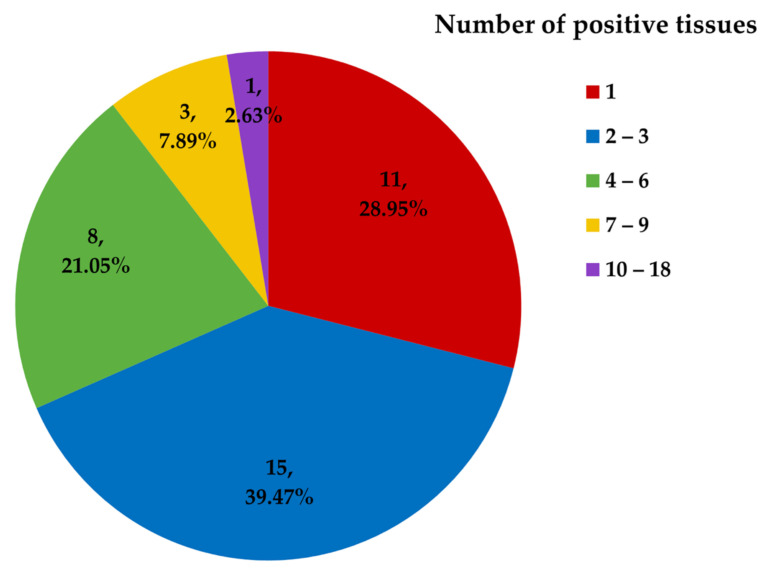
Classification of positive animals according to the number of tissues in which HV DNA was detected. Numbers of animals and percentage of all positive animals within each group are indicated.

Of the 38 positive animals, 27 (71.05%) were positive regarding more than one tissue, with each positive individual having an average of 3.18 positive tissues (121/38). This average number of positive tissues was 2.29 (78/34) after excluding four positive animals with more than six positive tissues. Positive animals were classified into five groups using the Jenks natural breaks algorithm in R software 3.2.2 (The R Foundation for Statistical Computing, 2014): 28.95% of infected individuals had one positive tissue, 39.47% had 2–3, 21.05% had 4–6, 7.89% had 7–9, and 2.63% had 10–18 (Figure 1).

The proportion of positive animals was similar for striped dolphins (28/35, 80.00%) and bottlenose dolphins (7/8, 87.50%) (Table 1). The proportion of samples that were positive was also similar for striped dolphins (70/694, 10.09%), bottlenose dolphins (20/149, 13.42%), and all three Risso’s dolphins (13/100, 13.00%); these proportions were similar to the study-wide average of 12.53%. In contrast, the one Cuvier’s beaked whale had an above-average proportion of positive tissues (18/23, 78.26%).

HV was present in several tissues more often than the overall average of 12.53% across all tissues (Table 1). With regard to those tissues of which more than 15 samples were analyzed, HV was present more often in the heart (21.05%); urinary bladder (20.59%); CNS (19.62%), which comprised the cerebellum (21.87%), brain (20.93%), pons (21.74%), lateral ventricle (18.75%), thalamus (15.00%), and cerebral cortex (one positive among three samples); pharyngeal tonsils (18.52%); skin (16.87%); suprarenal glands (16.13%); teeth and gingiva (12.82%); and cardiac blood (13.64%). HV was present at a frequency of almost 10% in the pancreas (9.68%), liver (9.52%), and blubber (9.52%). All 33 spleen samples tested negative.

The organ system that had the highest percentage of HV-positive samples (100 * HV-positive samples/number of samples evaluated) was the reproductive system (39.47%), followed by the nervous system (18.93%); the integument (14.40%); the endocrine (13.95%); and the circulatory (12.28%), urinary (11.63%), digestive (10.13%), respiratory (6.78%), and lymphatic (4.64%) systems. The musculoskeletal system was the less frequently infected (4.54%) (Table 1).

At an individual level, HV was equally prevalent in females (16/18, 88.89%) and males (20/25, 80.00%). However, some sex-related differences were observed at tissue level. Positive females contained a greater number of positive tissues (average, 4.37 (70/16); median, 3.5) than positive males (average, 2.25 (45/20); median, 2). Eight out of the 16 positive females had more than three positive tissues, whereas results of this nature were attained for only 3 out of 20 positive males.

**Figure 2:**
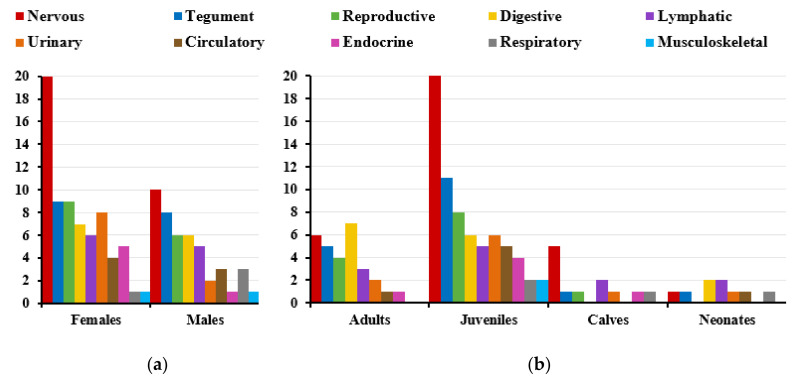
Number of tissues in each organ system that tested positive to HV DNA, stratified by sex (**a**) and age (**b**) of the animal. HV DNA was detected in 121 of 966 tissue samples (12.53%), which had the following distribution by sex and age: females (70/439, 15.94%), males (45/498, 9.04%), adults (29/377, 7.69%), juveniles (69/379, 18.21%), calves (12/83, 14.46%), and neonates (9/98, 9.18%).

Several sex-related differences were observed regarding tissue tropism (Figure 2a), primarily in the nervous system (females, 20 positive samples in 74 evaluated tissues (20/74) vs. 10/89 in males); urinary bladder (6/17 vs. 1/17); suprarenal gland (4/18 vs. 1/13); pancreas (3/16 vs. 0/15); reproductive system (9/17 vs. 6/21), mainly the external genitalia (genital mucosa and penis) (5/8 vs. 6/16); and liver (1/17 vs. 3/22).

**Figure 3:**
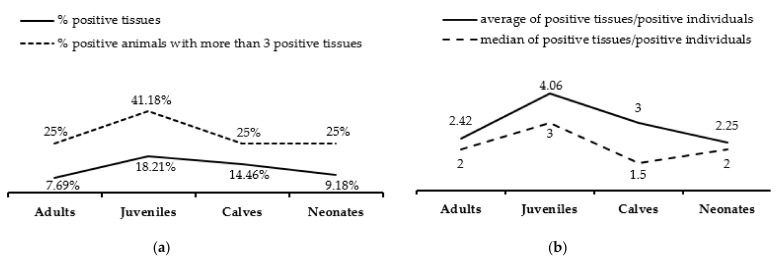
HV DNA detection stratified by age of the animal. (**a**) The percentage of all tissues that tested positive is shown, as is the percentage of infected individuals that had more than three positive tissues. (**b**) Average and median numbers of positive tissues per positive animal are shown.

Of the four age groups, HV prevalence was lower in adults (12/17, 70.59%) and neonates (4/7, 57.14%). Conversely, almost all juveniles (17/18, 94.44%) and all calves (4/4, 100%) were positive. Tissue samples from juveniles accounted for 57.02% (69/121) of all positive tissues, and infected juveniles had an average of 4.06 positive tissues (median 3). Nearly half of the juveniles (41.18%) had a higher number of positive tissues than the average of 3.18 calculated across all age groups (see Figure 3).

Several age-related differences were observed regarding tissue tropism (Figure 2b). The organ system most frequently infected in juveniles and calves was the nervous system, and this was the second most frequently infected organ system in adults. The system most frequently affected in adults and neonates was the digestive system. The tegument was infected more often in juveniles and in adults. The lymphatic system was infected more often in calves and neonates and to a lesser extent in juveniles. The reproductive system was the second most-often infected system in juveniles and the fourth most-often infected in adults. In contrast, it was found to be infected in only one of four calves and never in neonates (Figure 2b).

### 3.2. Herpesvirus RNA Detection to Assess Active Replication

GAPDH RNA was detected in 107 of the 121 samples containing HV DNA (88.43%); of these 107 samples, both RNA and DNA were detected in 54 (50.47%), indicating that the virus was actively replicating in these tissues. Of the 37 animals in which at least one tissue tested positive for both HV DNA and GAPDH RNA, 28 (75.68%) contained HV RNA in at least one tissue, indicating active replication.

**Figure 4:**
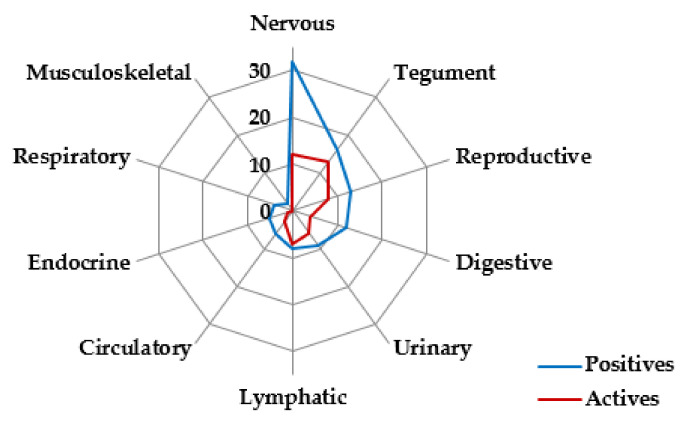
HV active replication in tissues of each organ system. Tissues positive for both HV DNA and GAPDH RNA were defined as the total set of positive tissues (“positives”), and the number of these positive tissues containing HV RNA was determined (“actives”). This provided an indicator of active replication. The results are shown for different organ systems.

The prevalence of HV RNA in different tissues was much lower than that of DNA. For example, only 12 of 32 nervous system samples (37.50%) contained RNA. RNA was also present in relatively small proportions of samples from the digestive (4/12, 33.33%), circulatory (3/6, 50.00%), endocrine (1/5, 20.00%), respiratory (0/4), and musculoskeletal systems (0/2) (Figure 4).

**Figure 5:**
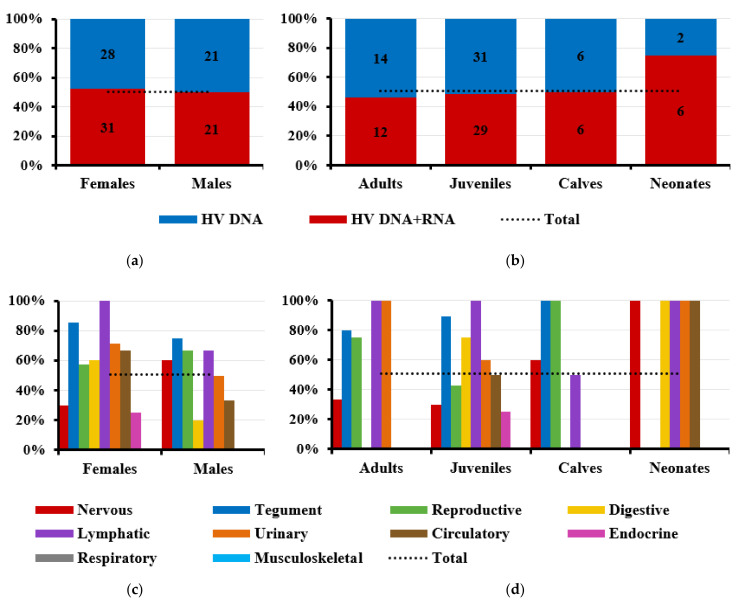
HV active replication in the tissues stratified by gender and age of the animal. Detection of active HV replication, defined as the presence of HV RNA, in tissues positive for HV DNA and GAPDH RNA. The dotted line indicates the proportion of tissue samples with active replication across ages and sexes (54/107, 50.47%). (**a**,**b**) Comparison of tissue samples that tested positive for only HV DNA or for both HV DNA and RNA (among all samples that were positive for GADPH RNA), stratified by sex (**a**) and age (**b**). Number of positive tissues is shown for each category. (**c**,**d**) Comparison of organ systems positive for only HV DNA or for both HV DNA and RNA (among all samples positive for GADPH RNA), stratified by sex (**c**) and age (**d**).

The proportion of tissues containing both HV DNA and RNA was similar in females (52.54%) and males (50%) (Figure 5a). However, sex-related differences were observed regarding active viral replication: very few samples of nervous or endocrine systems showed active replication in females; very few samples of digestive or circulatory systems showed active replication in males (Figure 5c).

Several age-related differences were observed regarding the prevalence of active HV replication. At the level of tissue samples, the prevalence of active viral replication in adults (46.15%), juveniles (48.33%), and calves (50%) was similar to the global prevalence across all ages (50.47%), while it was much higher in neonates (75%) (Figure 5b). At the individual level, however, the prevalence of active replication was lower than the global average for all age groups (75.68%), with the exception of juveniles (14/17, 82.35%). HV RNA was detected only in a few samples of nervous tissue, with the exception of neonates and calves. RNA was not detected in any samples of digestive tissue obtained from adults, despite the fact that this system was, according to the DNA analysis, the most frequently infected (Figure 5d).

### 3.3. Phylogenetic Analysis

**Figure 6:**
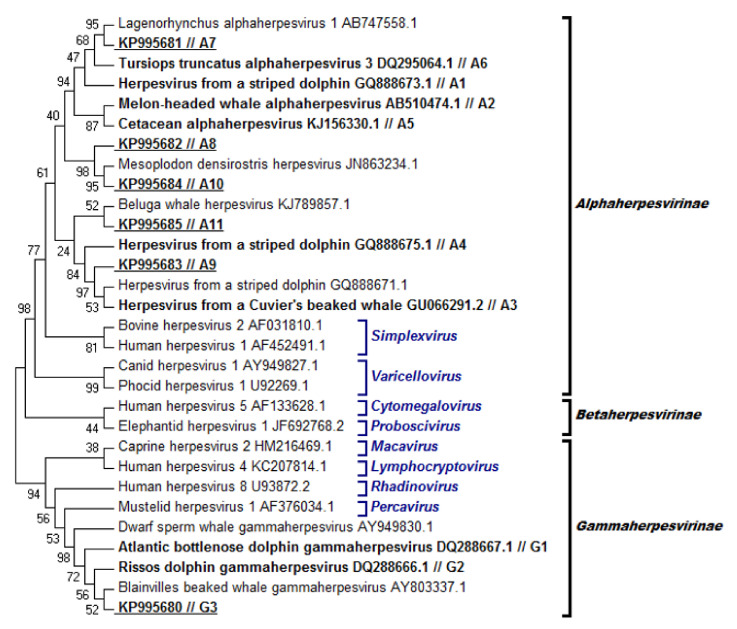
Phylogenetic analysis of HV based on partial sequences of the DNA polymerase gene from different host species. The phylogenetic tree was inferred from deduced amino acid sequences using the maximum likelihood method. Each sequence was named according to the virus and GenBank accession number; sequences registered as undefined HV were denominated as HV from + < common name of the host >. Sequences amplified in this study and the GenBank sequences to which they are identical are marked in bold type and labeled A1 to A11 (*Alphaherpesvirinae*) and G1 to G3 (*Gammaherpesvirinae*). Novel sequences are underlined and indicated together with their GenBank accession number. Sequences were assigned to HV subfamilies and genera according to Davison [28]. Betaherpesvirus sequences served as the outgroup with which to root the phylogram. Tree topology was inferred from p-distances using the maximum likelihood method. Topology reliability was tested by bootstrapping 500 replications, and the results are indicated at the tree nodes.

Amplicons, measuring 169, 181, or 193 base pairs (bp), were isolated from the 121 tissues that had tested positive for HV DNA. All sequences were compared to those in GenBank and were found to be identical, or the most closely related, to HV sequences isolated from cetaceans. All sequences were aligned with sequences from other HV in order to construct a phylogenetic tree (Figure 6). The reliability of the alignment and, therefore, of the resulting tree, was judged to be adequate given that the average amino acid p-distance was 0.5114, which was lower than the prespecified threshold of 0.8.

This phylogenetic analysis revealed that 42 of the 121 positive sequences belonged to the *Alphaherpesvirinae* subfamily and the other 79 to *Gammaherpesvirinae*. Fourteen unique sequences were identified and named using consecutive numbering and by referring to the HV subfamily: sequences A1–A11 belong to *Alphaherpesvirinae*, while G1–G3 belong to *Gammaherpesvirinae*. Six of these sequences proved to be novel and were deposited in GenBank with the following accession numbers: sequence A7, KP995681; A8, KP995682; A9, KP995683; A10, KP995684; A11, KP995685; and G3, KP995680. Most sequences belonged to *Gammaherpesvirinae*, while the *Alphaherpesvirinae* sequences had a greater variation (Figure 6).

Of the 38 animals that tested positive for HV DNA, 11 (28.95%) had only one positive tissue. Of these animals, 10 were positive for gammaherpesvirus (GHV) and only 1 for alphaherpesvirus (AHV). The remaining 27 individuals (71.05%) had at least two positive tissues; 4 individuals were positive only for AHV (4/27, 14.81%), 9 for both AHV and GHV (9/27, 33.33%); and 14 only for GHV (14/27, 51.85%). Eleven of these 27 animals (40.74%) were infected with at least two HV strains.

**Figure 7:**
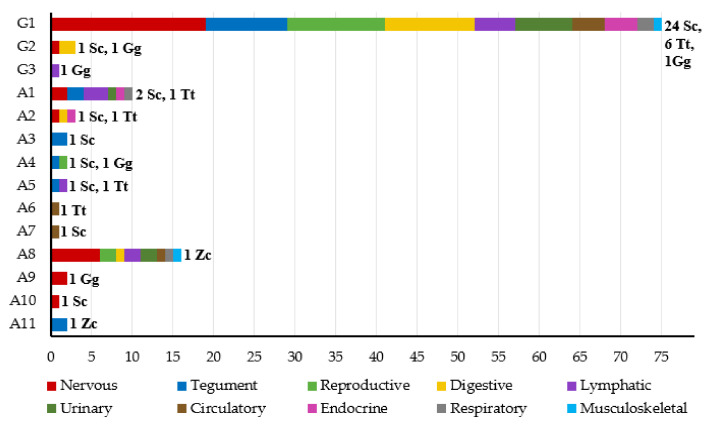
Frequency of detection of each unique HV sequence in tissues of each organ system. Sequences amplified in this study were assigned identifiers from A1 to A11 (*Alphaherpesvirinae*) and G1 to G3 (*Gammaherpesvirinae*). The number of individuals of each species in which the sequence was found is shown. Sc, *Stenellacoeruleoalba*; Tt, *Tursiops truncatus*; Gg, *Grampus griseus*; Zc, *Ziphius cavirostris*.

Most amplified sequences corresponded to the following: G1, found in 75 of 121 tissues (61.98%) and in 31 of 38 individuals (81.58%); A8, found in 16 of 121 tissues (13.22%) and in only one individual (2.63%); or A1, found in 10 of 121 tissues (8.26%) and in three individuals (7.89%). The other unique sequences were found in only one to three tissues from one to two individuals. Most sequences were amplified from different tissues and species, suggesting no restriction to a given tissue or species (Figure 7). Interestingly, only sequence G1 was detected in liver samples.

### 3.4. Gross Lesions and Histopathological Study

**Figure 8:**
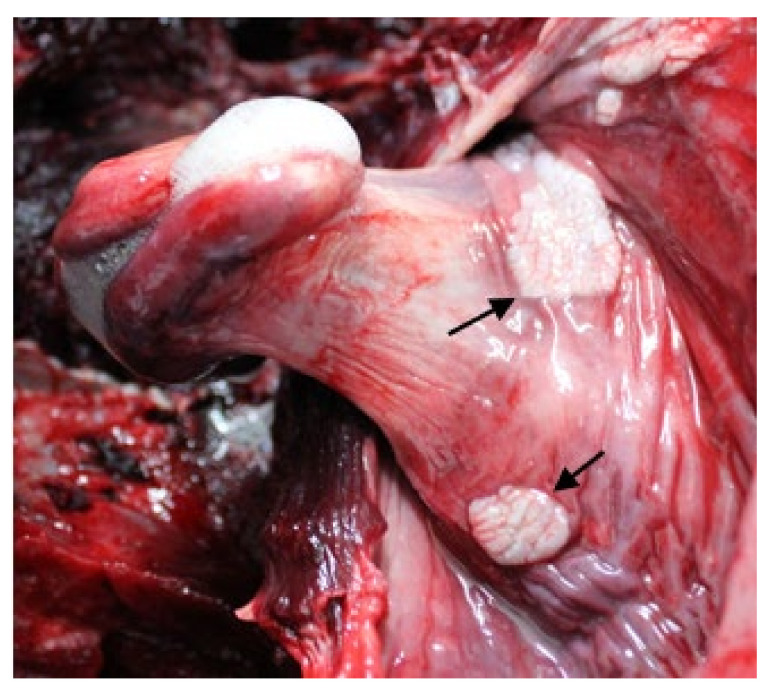
Risso’s dolphin, oropharynx, macroscopic image. Larynx with multifocal, discrete, raised, white plaques with rough surface (arrow).

The gross lesions of HV-positive organs were mostly nonspecific, with a predominance of congestion. Relevant lesions were observed only in the reproductive system, the esophageal mucosa, and the integument. The only lesion detected in the genital mucosa comprised two whitish proliferative structures of 1 cm in length, which stood out on the mucosa around the vagina in the genital fissure. The lesions of the esophageal mucosa consisted of ten white structures that were papillomatoid in shape and of varying sizes (between 2 and 5 cm in length, 1–2 cm in width, and 0.2–0.3 cm in relief), with irregular borders, around the larynx (Figure 8). Lesions were detected on the skin of three individuals: dark gray punctate lesions (0.1 cm); dark circular lesions with a slight depression; and an oval lesion (1.5 × 1 cm) with a slight relief, irregular surface, and no change in color, craniolateral to the blowhole.

Histopathology was possible in 26 of the 38 individuals, in which HV DNA was detected in one or several tissues and organ systems. The majority of cellular changes and lesions in these tissues and organs could not be specifically or uniquely associated with an active HV infection. Most were probably caused directly by concomitant bacterial or parasitic or viral infections. Other lesions were chronic and non-specific, and could not, therefore, be associated with an identifiable cause.

**Figure 9:**
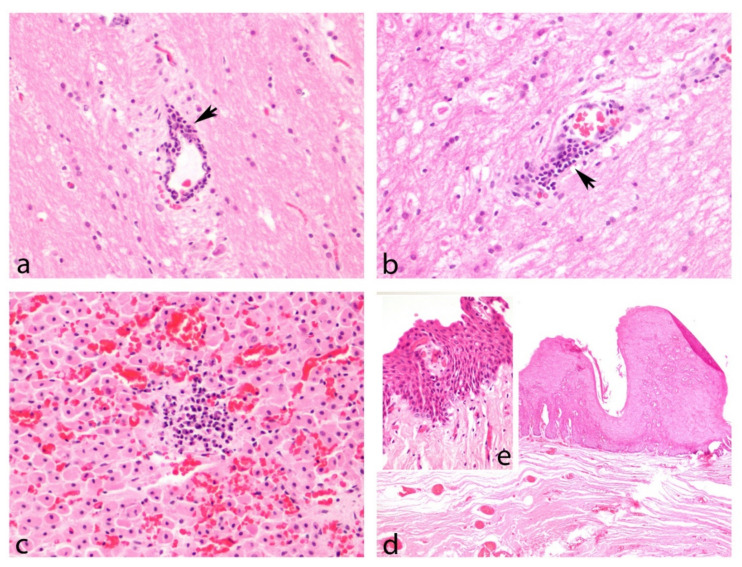
Histopathological images of representative lesions. Hematoxylin–eosin stains. (**a**,**b**) Striped dolphin, brain, 40×. Lymphoplasmacytic meningoencephalitis associated with GHV infection in the CNS. Perivascular cuffs of lymphocytes, plasma cells, and few histiocytes (arrow) in cerebral white matter. (**c**) Striped dolphin, adrenal gland, 40×. The zona fasciculata of the adrenal cortex contains a small, nodular infiltrate of lymphocytes and plasma cells, which separate and extend between adjacent spongiocytes. Diffusely, the cortical sinusoids are markedly congested. (**d**) Risso’s dolphin, penis, 4×. A well demarcated area of mucosa is markedly hyperplastic, containing numerous layers of squamous epithelium that lay over an irregularly folded basement membrane. (**e**) Detail of the previous lesion at 40×, showing basal cells piled along the folded basement membrane and mild spongiosis of the cells in the middle layers.

GHV DNA and concomitant lesions were detected in the CNS, lung, urinary bladder, genitalia, tegument, blubber, skeletal muscle, liver, esophagus, and adrenal gland (Table 2). Samples obtained from the CNS of five individuals that were positive for the G1 subfamily sequence had non-suppurative encephalitis (Figure 9a,b), while seven animals had non-suppurative meningitis. In one animal, the CNS samples that were positive for the G2 sequence had a mild leukocyte infiltration of the meninges with congestion and a mild hemorrhage. In another individual, the esophageal mucosa contained multifocal hyperplastic plaques, with mild ballooning degeneration of the mid-layers and piling of the basal cells. This lesion corresponds to the structures shown in Figure 8. This tissue was positive for the G2 sequence. Mild multifocal, lymphoplasmacytic adrenalitis (Figure 9c) was observed in one sample that was positive for the G1 sequence.

The only sample of lung that tested positive for GHV had concomitant lesions. The pulmonary parenchyma contained multifocal bronchi with sloughed cells, and the surrounding interstitium was expanded by infiltrates of lymphocytes that extended into the alveolar septae. The adjacent alveoli contained foamy macrophages and protein fluid.

In one dolphin, the vaginal mucosa that tested positive for GHV contained a plaque of hyperplastic epithelium, and the underlying mucosa was infiltrated by small aggregates of lymphocytes and plasma cells. This lesion corresponded to the proliferative structure detected at necropsy. Another female individual also had mild lymphoplasmacytic infiltrates in the submucosa of GHV-positive vaginal tissue.

Positive tegument samples contained foci of epithelial hyperplasia; hyperkeratosis; rare eosinophilic intranuclear inclusions; and, to a lesser extent, hydropic degeneration of the keratinocytes, intravascular leukocytosis, individualized necrotic cells, and mild pigmentary incontinence. Two of these hyperplastic dermatitis corresponded to the circular and oval lesions described in the gross lesions.

Liver samples from two individuals that tested positive for the G1 sequence contained suppurative hepatitis with intralesional bacteria and a chronic lymphoplasmacytic and proliferative cholangiohepatitis with fibrosis.

In one animal, samples of the urinary bladder that was positive for the G1 sequence contained chronic lymphoplasmacytic cystitis, with areas of erosion and ulceration.

AHV DNA and concomitant lesions were detected in the lymph nodes, skin, genitalia, lung, CNS, heart, and kidney (Table 2). Specifically, subfamily A1, A2, A8, and A9 sequences were detected in the CNS, with concomitant lesions mainly owing to vascular disorders such as vasogenic edema, congestion, and haemorrhaging. Mild intravascular leukocytosis or increased numbers of meningeal glial cells were also observed.

The lungs from two individuals that tested positive for sequences A1 and A8 had multifocal interstitial bronchopneumonia and pyogranulomatous pneumonia with intralesional nematodes, and severe subacute multifocal fibrinonecrotic and suppurative bronchopneumonia, respectively. The latter contained intralesional bacterial colonies.

Interestingly, lesions in the genitalia were found only in penile mucosa infected with the subfamily A4 sequence. The mucosa was variably and multifocally hyperplastic, with mild hydropic degeneration and occasional eosinophilic intranuclear inclusions. Multifocally, the mucosal epithelium was separated from the submucosa by clefts of colorless space containing individualized necrotic cells and hemorrhage, and the underlying submucosa was sprinkled with perivascular lymphoplasmacytic infiltrates (Figure 9d,e).

Tegument samples infected with subfamily AHV, A3 and A4 sequences, contained areas of epithelial hyperplasia, mid-layer hydropic degeneration, subepithelial clefts, piling and anisocytosis of the basal layer, and occasional intranuclear and eosinophilic inclusions. One of these hyperplastic dermatitis corresponded to the punctate lesions described above.

Several lymph nodes that were positive for sequences A1 and A8 had moderate lymphocyte depletion, with thinned cortices and a few small follicles containing apoptotic and phagocytosed lymphocytes.

One sample of myocardium that was positive for sequence A7 contained multifocal acute myodegeneration and mild leucocytosis.

Chronic interstitial lymphoplasmacytic nephritis was observed in the kidney samples obtained from two individuals, which were positive for A1 and A8 sequences.

### 3.5. Herpesvirus and Morbillivirus Coinfection

Of the 47 individuals studied in this research, nine striped dolphins (19.14%) were diagnosed with CeMV by real time RT-PCR, comprising three adults, four juveniles, and two calves. All nine also tested positive for HV DNA, signifying a prevalence of coinfection with HV of 100% in those animals that were positive for CeMV. However, both viruses were not detected simultaneously in the same tissues, with the exception of the brain from a juvenile. HV prevalence in this subset of nine striped dolphins was similar to the prevalence in the entire study population, at both the individual and tissue levels. The nine striped dolphins had HV DNA in 1–4 tissues (average, 2.33), which was similar to the average of 2.29 for the 34 cetaceans in the study, of which fewer than five tissues tested positive for HV. The proportion of HV-positive tissue samples obtained for the nine striped dolphins (11.67%) was similar to that obtained for all the striped dolphins (10.09%) and all the cetaceans (12.53%) analyzed in this work. Moreover, the proportion of tissue samples obtained from the nine striped dolphins that was positive for active HV replication (47.62%) was similar to that obtained for all the animals in the study (50.47%).

The animal with cerebral tissue that tested positive for both viral agents had a moderate diffuse lymphoplasmacytic meningoencephalitis with perivascular cuffing, gliosis, satelitosis, and mild neuronophagia.

The lesions associated with CeMV were described by Rubio-Guerri et al. [60] and consisted mainly of non-suppurative encephalitis, broncointerstitial pneumonias, and nephritis.

## 4. Discussion

The present study reveals a high prevalence (80.85%) of HV in free-ranging cetaceans off the coast of the Valencian Community. This is the highest HV prevalence ever reported for marine mammals. The prevalences of HV infection previously reported in cetaceans are as follows: less than 10% in Japan [34], Brazil [22], and on the Portuguese and Galician (Spain) coast [20]; 14.45% in the Canary Islands (Spain) [30]; 62.5% on the Mediterranean Spanish coast [24]; and 78.57% in Cantabria (Spain) [21]. The present study also examines perhaps the largest variety and number of tissue samples ever published for stranded cetaceans. This extensive analysis has allowed us to show that most of the animals were infected in multiple tissues, and that the virus was actively replicating in approximately half of the infected tissues. The true HV prevalence in our study may be even higher, given that five of the nine animals found to be negative for HV were tested in only 4–7 tissues, which is much lower than the global average of 20.55 tissues tested per individual. This increases the risk of false negative determinations.

Eleven out of the 27 (40.74%) animals analyzed in our study had coinfection regarding least two HV strains. Concomitant infection with different HV strains has previously been described in cetaceans [9,15,20,24]. In particular, the only Cuvier’s beaked whale included in this study attained the highest number of positive samples (*n* = 13), and was coinfected by two different viral variants (A11 on the skin and A8 in the other positive organs) (Table 2). Coinfections by two different sequences of AHV have been previously reported in the *Ziphiidae* family [30].

The greatest proportions of HV-positive samples in our study occurred in the same tissues in which most previous reports have identified HV in cetaceans: the reproductive system [6,8,9,11,16,17,18,20,21,23,31,32], the CNS [4,10,15,17,21,23], and the tegument [2,3,5,6,7,9,15,17,21,22]. The bias that could have arisen owing to the number of samples taken from each system was eliminated by studying the tropism as a proportion over the number of samples analyzed, rather than in absolute numbers (for example, CNS samples were taken from various regions of the same individual). This signifies that, although the CNS had the highest number of positives, the organ system that had the greatest proportion of HV-positives was the reproductive system.

The HV presence in our study was higher in females than in males, particularly in the nervous, urinary, endocrine, and reproductive systems. In a study carried out with Beaked Whales stranded in the Canary Islands (Spain), the prevalence in females was also higher than in males (5/8, 62.5%) [30]. However, the prevalence was higher in males in a mass stranding in Cantabria (Spain) (9/11, 81.82%) [21], and in two studies carried out in Brazil and Japan (2/3 animals with identified sex, 66.67%) [22,34]. In another study carried out in Portugal, the proportion of males and females infected with HV was, meanwhile, 50% (7/14) [20]. With regard to the pinnipeds, nasal swab-based tests revealed a higher HV prevalence in female Hawaiian monk seals [61], in addition to which a higher seroprevalence was found in female harbor seals and sea lions [62]. However, urogenital swab-based tests for otarine herpesvirus-1 showed a higher prevalence in males [63]. Further studies are required in order to understand the influence of an individual’s sex on HV infections.

The HV prevalence in the present study varied strongly with age; it was the highest for juveniles and calves, and the lowest for neonates. In fact, juveniles had the highest proportion of positive tissues, along with the largest number of positive tissues per animal. Similar results were reported for Hawaiian monk seals, for which the proportion of positive samples was the greatest for subadults and the smallest for pups [61]. It has been suggested that there is a positive correlation between the age of walruses and the probability of their serum containing anti-HV antibodies [62]. This trend was partially observed in the present study, although the prevalence of HV was lower in adults than in juveniles, at the level of both individuals and tissue samples. In previous studies in cetaceans, HV infection has been described in eight individuals, all of them adults, in the Canary Islands [30]; in Brazil, the infection was reported in two adults, one juvenile and one calf [22], while in Japan, HV infection was diagnosed in two infants, one neonate and one adult [34]. Previous studies concerning HV in cetaceans when considering the age of the infected individuals have been based on a low number of HV-positive individuals (less than 10), while in our study, 38 positive animals were studied. However, more research work taking into account a greater number of cetaceans will be necessary to elucidate whether age acts as a risk factor for HV infection in these animals.

The neonates in our study had the lowest prevalence of HV based on DNA, yet they contained the highest proportion of HV-positive tissues in which RNA was also detected, thus indicating active viral replication. This may reflect more recent infection before becoming latent; it may also reflect relatively low immunocompetence.

In the present study, we assumed that the presence of HV RNA indicated active replication, as mRNA with the HV DNA polymerase sequence was detected, as previously described in HV infections in marine mammals [57]. Assuming that the absence of RNA indicates latent infection, it is interesting to note that RNA was found in only 12 of 32 HV-positive samples obtained from the nervous system (37.50%). This may reflect the ability of HV to establish a lifelong latent infection in neurons [25,26]. However, the absence of RNA does not definitively indicate latent infection, as it can also arise from abortive or very early infection or because RNA levels are too low to be detected. Future work should examine HV latency in cetaceans in more detail.

The present study detected 14 unique sequences from the HV DNA polymerase gene, one of which (G1) occurred significantly more often than other sequences—it was found in 81.58% of positive individuals. All sequences were found to be identical, or most closely related, to GenBank sequences of HV isolated from other cetaceans. This supports previous observations that the phylogenetic branching of *Herpesviridae* resembles that of their hosts [19,57,64], consistent with host–virus co-evolution.

An examination of the subset of animals infected with CeMV (nine striped dolphins) showed that 100% of cetaceans infected with CeMV were coinfected with HV. Furthermore, the proportion of positive tissues and active replication was similar between that subset and the entire study group. As occurred with this prevalence of HV in the CeMV-infected animals analyzed herein, an analysis of eight striped dolphins from a 2007 CeMV outbreak showed that 62.5% of the individuals infected with CeMV were also infected with HV [24]. HV prevalence may have been underestimated in the previous study, because only 1–7 tissues per animal were tested for HV. In another study carried out on the Portuguese and Galician coast, it was observed that, of the 70 dolphins studied, 10 had HV infection, and 2 of these animals were also coinfected with CeMV [20]. In our study, all the CeMV positive animals were also HV-positive, but coinfection was found only in the same tissue in a single sample, from the cerebellum. Something similar occurred in a study carried out in the Canary Islands, in which the only coinfection by these two agents was reported in the brain of one individual [15]. The coinfected cerebellum of this study had a lymphoplasmacitic meningoencephalitis, which can be caused by both agents.

Furthermore, lesions compatible with CeMV were found in some of the HV-infected tissues of these coinfected animals, despite the fact that the molecular diagnosis of CeMV was negative. Examples of this are the chronic interstitial pneumonia found in the striped dolphin stranded on 13 March 2011, or the lymphoplasmacytic cystitis with intracytoplasmic inclusion bodies (IBs) diagnosed in the striped dolphin stranded on 16 March 2011. These animals had two and six CeMV-positive tissues, respectively, including the kidney, with a chronic nephritis, in the second [60], which are probably more closely related to the action of CeMV than to HV. It is possible that CeMV genetic material was not detected in these tissues because the animal overcame the infection in these organs, but the tissue damage persisted. Something similar occurred with the striped dolphin stranded on 26 January 2012. Although this animal did not attain a positive molecular diagnosis for CeMV in any organ, it also had interstitial bronchopneumonia, which is morphologically suggestive of CeMV infection. In addition, there was a higher than average presence of nematodes, which is often observed to be concomitant with immunosuppresion. Both HV and CeMV are known to suppress the immune system [12,65] and could, potentially, be the underlying cause. Studies on a larger scale should examine CeMV and HV coinfection in greater detail. As both viruses cause immunosuppression, coinfection may be an important factor to take into account when using cetaceans as marine ecosystem sentinels.

With regard to its pathological significance, HV can be highly ubiquitous, but its ability to cause disease and mortality in cetaceans is unclear and may vary depending on the age, immune status, or stress level of an individual [4,13].

In cetaceans, CNS damage has been described only when associated with the AHV [4,10,15,23], and the main lesion typically associated with infection consists of non-suppurative meningoencephalitis [4,10,15,23]. In our study, we detected mild to severe encephalitis, meningitis, or non-suppurative meningoencephalitis in eight individuals, all associated with GHV infection. In addition, in one individual, coinfection with the G1 and A2 sequences was detected in the brain and meninges, respectively. The affected individuals were juveniles (*n* = 5), calves (*n* = 2), and adults (*n* = 1). Other more nonspecific signs, such as hemorrhage and/or congestion (19/24), vasculitis (3/24), and edema (3/24), were also recorded.

CNS infection by GHV has been described in cetaceans (both odontocetes and mysticetes) [17,21], but associated lesions have never been described. In the present case series, encephalitis or meningoencephalitis was detected in GHV-positive samples. In fact, the most severe encephalitis and meningitis were associated only with sequence G1, which belongs to the GHV subfamily. To the best of our knowledge, this is the first description of CNS lesions in GHV-positive samples in odontocetes. GHV have been linked to non-suppurative encephalitis and other CNS lesions in other mammal species. A broad spectrum in the neuropathological phenotype has been described in sheep, ranging from no injury to severe injury [66]. Inflammatory changes associated with this infection were found to be more commonly moderate or severe; vasculitis was the most common pathological presentation observed in the neuroparenchymal and meningeal vessels; other signs such as neurophagia or gliosis were present; and, in some cases, there were also indicators of vascular damage and an increased permeability of the blood–brain barrier (hemorrhages, loss of perivascular proteins) [66]. GHV has been suggested to be the causative agent of non-suppurative encephalitis in black bears [67], while here we report the presence of meningitis and encephalitis in GHV-positive samples in cetaceans (six striped dolphins and one bottlenose dolphin). However, non-suppurative inflammations in the CNS have also been associated with other agents, such as CeMV, *Brucella ceti*, or *Toxoplasma gondii* [15,68]. Although a co-infection between HV and CeMV was found in only one sample, the presence of other agents such as *B. ceti* and *T. gondii* that may have participated in the pathogenesis of the lesions cannot be ruled out. Unfortunately, this information is unavailable. Future studies should analyze whether GHV is able to cause damage to the CNS in cetaceans.

With regard to the skin, HV infection in cetaceans has been associated with different types of dermatitis, including proliferative, fibrinosuppurative, and necrotizing dermatitis [2,3,7,22,69,70]. In addition, white-fringed, as well as pale and cloudy, macroscopic lesions, associated with HV, have also been reported without a histopathological description [71]. In this study, we have obtained a range of skin lesions in HV-positive samples, from nonspecific lesions to lesions that are typical of HV infection, and one case in which lesions were absent. Intranuclear amphophilic inclusions were occasionally observed in a female bottlenose dolphin with prior erysipelas [72], together with epithelial cell necrosis. Epidermal necrosis is consistent with HV [2,3]. Epithelial hyperplasia with eosinophilic intranuclear inclusions, keratinocyte vacuolization, or hydropic degeneration, along with the multifocal separation of the epidermis and dermis, have been observed in three striped dolphins, two of which also had hyperkeratosis. Dermoepidermal separation is a consequence of hydropic degeneration [73], and hydropic degeneration has previously been associated with HV in cetaceans [22]. In another individual, the only findings were the presence of eosinophilic intranuclear inclusions; mild intravascular leukocytosis; and, in the hypodermis, the presence of a granuloma with parasitic structures (possibly nematodes) inside it. It was not known whether the intranuclear inclusions were of a viral nature or if and how they were associated with HV. However, in the scenario that these inclusions were not viral, the absence of viral inclusions does not dismiss HV infection, as IBs are rarely observed after day 7 post infection [57,74]. In the remaining individuals, one had no lesions, and in the other, the lesions were focally extensive pigmentary incontinence with the presence of lymphocytes, and the slight vacuolization of keratinocytes. Although hydropic degeneration and epidermal hyperplasia are also caused by poxvirus [75], these lesions are accompanied by large characteristic eosinophilic intracytoplasmic IBs [57,75]. Lesions compatible with HV were detected in these three animals, although intracytoplasmic IBs were also observed. Although HV DNA was detected and the morphology of the lesions was, in many respects, suggestive of an HV infection, this interpretation must be viewed with caution because of the similarities with poxvirus. Unfortunately, this information is unavailable. Nevertheless, the existing possibility of HV and poxviral infection, together with the presence of parasitic granulomas in the skin, exemplify the frequency of occurrence of concomitant infections associated with the presence of HV. This circumstance highlights the possibility of underlying immunosuppression, which is in turn associated with HV infection [12].

In our study, several different lesions were found in genital samples that tested positive for HV PCR, while one individual with a positive detection of HV lacked lesions. The three main lesions were multifocal lymphoplasmacytic inflammation (three individuals), epithelial hyperplasia (two individuals), intranuclear inclusions (two individuals), vacuolation of epithelial cells (one individual), and necrotic-apoptotic cells (one individual). One individual also had intracytoplasmic IBs. The presence of lymphoplasmacytic aggregates in the genital submucosa of cetaceans is, within normal limits, considered to be mucosal associated lymphoid tissue (MALT) and is not necessarily indicative of infection [76]. Epithelial hyperplasia is one of the main findings associated with HV in cetaceans, especially in the genital mucosa [8,11,18,23,32,69]. This hyperplasia is often associated with characteristic eosinophilic or amphophilic intranuclear IB [11,18,23,32,69]. These differ from the intracytoplasmic eosinophilic inclusions that are indicative of poxvirus, which may also cause hyperplastic lesions. Proliferative genital lesions in cetaceans can also be caused by papillomavirus [31,77], and co-infections between these two agents have been described in dolphins [31,78]. In the case of this type of lesions, such as lymphoplasmacytic and hyperplastic vaginitis of the bottlenose dolphin stranded on 14 July 2010 or the proliferative balanitis of the Risso’s dolphin stranded on 11 December 2011, this agent should, therefore, be taken into account in a differential diagnosis. The hydropic degeneration of epithelial cells has also been described in other genital lesions associated with HV in odontocetes [11,16]. In ruminants, Bovine herpesvirus 1 infection has been associated with the presence of epithelial necrosis, lymphocytic infiltration, and the appearance of intranuclear IBs in the genital mucosa [76]. Necrosis, intranuclear inclusions, and lymphocitic infiltration were found in one of the studied Risso’s dolphins. HV infections without associated lesions in cetacean genital organs have also been described previously [79].

With regard to the digestive system, the most striking lesions were the papillomatoid-shaped structures in the esophagus, around the larynx, which histologically corresponded to focally expansive irregular acanthosis (Figure 8). Similar plaque-like mucosal proliferations around the larynx have been associated with GHV in Rough-Toothed Dolphins (*Steno bredanensis*) [80]. Other lesions affecting the mucosa of the upper digestive tract, particularly in oral mucosa and the tongue, have also been associated with HV infection in cetaceans [9,16,22,80]. Moreover, erosive and ulcerative lesions in the distal region of the esophagus associated with GHV infection have been described in sea lions [29]. HV is also capable of causing injuries to the upper digestive system, including the esophagus, of ruminants. These lesions in cattle and goats are usually erosions or foci of inflammation, and usually occur in animals of less than 2 months of age [74,81]. The Risso’s dolphin with the papillomatoid structures in the esophagus and larynx was an adult male, and its lesions consisted of well-delimited foci of non-ulcerated hyperplastic mucosa, similar to the plaques observed in the same animal’s penis, despite the fact that the HV subfamilies and sequences identified in each location were different (G2 in the esophagus and A4 in the penis). However, similar esopharyngeal lesions have also been found in harbor porpoises, and are associated with cetacean papillomavirus [82]. As this animal also had lesions compatible with papillomavirus in the mucosa of the penis, a co-infection with this agent cannot be ruled out.

Hepatic lesions associated with HV infection have been described in seals; that is, hepatitis, dystrophic degeneration, and massive coagulation necrosis [83,84]. The only lesions found in the livers of the animals studied herein were bacterial hepatitis and chronic cholangiohepatitis with fibrosis and bile duct hyperplasia, which are associated with trematode migrations similar to those described previously [85]. In a similar case, periductal fibrosis with lymphoplasmacytic pericholangitis associated with a severe parasitic infection in an HV-positive liver sample obtained from a Blainville’s beaked whale was also described [30]. The role of HV as an immunosuppressive agent [12,35,37] in concomitant infections has yet to be elucidated in this location.

The main lesion found in the kidneys was chronic lymphoplasmacytic nephritis. This finding was non-specific, and has been associated with ascending bacterial infections, leptospirosis, and parasitic migrations [86]. Membranous glomerulonephritis, multifocal interstitial lymphoplasmacytic nephritis, and acute multifocal necrotizing tubulointerstitial nephritis have been described in HV infections in beaked whales [12,13,30]. In the case of the lesions in the urinary bladder, these were nonspecific and were probably associated with other infectious and/or parasitic agents.

With regard to the lymphatic system, severe diffuse coagulative necrosis and fibrinonecrotic vasculitis with prominent thrombi in the lymph nodes and spleen, which are associated with HV, have been previously described [12]. This necrosis in lymphoid tissue associated with HV infection has also been described in goats [87]. Evidence of overt lymphoid necrosis was not observed in any of the tissues in this study. However, two of the lymph nodes examined in the Cuvier’s Beaked Whale (pre-escapular and pulmonary) had moderate diffuse lymphoid depletion with apoptotic cells. The fact that more than one lymph node was affected with this type of lesion, in a case of systemic infection by HV in a juvenile animal, together with the presence of apoptosis, was compatible with an active viral infection, consistent with HV. Histiocytosis was described in the other affected individual, indicating the presence of an antigenic stimulus. The presence of multinuclear giant cells and macrophages may be associated with the presence of CeMV or parasites.

Three types of pneumonia were found in the respiratory systems of the HV-positive animals: a severe subacute multifocal suppurative and fibrinonecrotic aspiration pneumonia associated with laryngeal necrosis; pyogranulomatous pneumonia caused by nematodes; and multifocal lymphocytic broncointerstitial pneumonia associated with bacterial colonies. HV has been associated with interstitial pneumonia in dolphins [6], and a viral origin has been suggested in a lung sample with a positive PCR for HV and a histopathological diagnosis of pneumonia with possible secondary bacterial infection [20]. Various types of pneumonia have also recently been described in HV-positive lung samples, although they were considered to be additional findings: mild multifocal parasitic bronchopneumonia with the presence of intraluminal nematodes, and mild multifocal interstitial bronchopneumonia [30]. Moreover, mild multifocal interstitial pneumonia has been described in a coinfection with CeMV [14]. HV can cause severe interstitial pneumonia in other marine mammals, such as seals [83,84]. In cattle, HV has been reported to alter mucociliary clearance and reduce the alveolar macrophage function, thus making them predisposed to bronchopneumonia caused by opportunistic bacterial pathogens [74]. HV could be considered the inciting cause of the lymphocytic broncointerstitial pneumonia with secondary bacterial infection. Interstitial pyogranulomatous bronchopneumonia associated with nematodes is a fairly common finding in cetaceans [88].

In this study, we report bilateral lymphoplasmacytic interstitial adrenalitis, and both adrenal glands were also infected by a GHV (G1 sequence). A similar case has previously been documented in a bottlenose dolphin, for which similar lesions associated with the detection of HV in the adrenal gland were described [89]. However, in cases of adrenalitis in cetaceans, a differential diagnosis should be made with erratic parasitic trajectories, as it has also been seen to produce this (authors’ unpublished observations). In beluga whales, vasculitis and necrosis in the adrenal glands have been associated with HV [70]. Another HV, porcine herpesvirus 1, also affects the adrenal glands [90].

The lesions detected in the heart and muscle were nonspecific and not associated with any infection.

Gross lesions were nonspecific, with the exception of those detected in the genital mucosa, pharynx, and skin.

With regard to the presence of HV mRNA, the most severe meningoencephalitis corresponded to the presence of mRNA, which could suggest a relationship between replication and inflammation. However, there were other samples in which, despite having detected HV mRNA, no significant lesions were found. Specifically, in three cases (a bottlenose dolphin, a striped dolphin, and a Cuvier’s baked whale), mRNA was detected in CNS without lesions. In the bottlenose dolphins, mRNA was also detected in the lymph nodes, adrenal gland, and mammary gland, with no lesions. This could have been owing to an abortive infection, controlled by the host’s immune system, or because this virus is more adapted to this species of dolphin. Most HVs are considered to have evolved in association with a single host species [91], thus reducing the pathological impact on the species with which they have co-evolved. In fact, the G1 sequence was associated only with two lesions in the bottlenose dolphins (lymphoplasmacytic and hyperplastic vaginitis and hyperplastic dermatitis), and it is not, therefore, possible to rule out that the lesions were caused by other agents not analyzed in this study (such as poxvirus or papillomavirus). Moreover, the G1 strain was first identified in a bottlenose dolphin on the coast of North Carolina. The other two cases are associated with sequences of the AHV subfamily. Interestingly, in the two individuals in question, RNA was detected only in the CNS, despite having detected HV in different tissues and organ systems. The HVs of the AHV subfamily establish their latency in neurons, and are reactivated in cases of stress and immunosuppression, signifying that one possible explanation is that the virus reactivated as the result of the stress that the animal underwent upon becoming stranded, without time for cell damage to occur. The asymptomatic replication of HV has been reported previously [92].

The lack of HV RNA in DNA positive tissues could suggest an abortive infection or very low levels of RNA in those tissues.

Samples of the uterus, mammary gland, skeletal muscle, and pancreas were within normal limits in all of the animals examined histologically. Although some of the lesions in tissues resembled those caused by HV, the majority were not specific to this agent and could have been related to other bacterial, parasitic, or other viral etiologies, despite having detected viral DNA or even mRNA. However, the role of HV as an immunocompromising agent with the potential of favoring concomitant or secondary infections has yet to be elucidated in marine mammals.

## 5. Conclusions

This analysis of cetaceans stranded off the Valencian coast suggests an HV prevalence of over 80%. The virus appears to produce a moderate degree of tissue tropism in cetaceans, and several tissue types are primarily affected: the heart, urinary bladder, CNS, pharyngeal tonsils, skin, suprarenal glands, teeth-gingiva, and cardiac blood. The organ system with the highest proportion of HV-positive samples was the reproductive system, followed by the nervous system and the tegument, endocrine, circulatory, urinary, digestive, respiratory, and lymphatic systems. The musculoskeletal system was infected least often.

Half of the HV-positive tissue samples showed evidence of active replication. Active replication was never detected in the respiratory or musculoskeletal systems, and it was rarely detected in the nervous, digestive, circulatory, and endocrine systems.

Females contained a greater number of positive tissues. Calves and juveniles had the highest prevalence of infection, with juveniles having the highest proportion of positive tissues and the largest number of positive tissues per animal. In contrast, neonates had the highest prevalence of active replication.

The most severe lesions in the CNS in samples with HV infection were associated with the presence of mRNA. Furthermore, this is the first time that CNS lesions have been identified in tissues with GHV infection in cetaceans.

This is, to the best of our knowledge, the first systematic HV DNA and RNA study on a wide collection of free-ranging cetacean tissues. The evaluation of HV prevalence in cetaceans may provide useful insights into marine mammals’ health.

## Figures and Tables

**Table 1:** HV detection in cetaceans at tissue level, in addition to being stratified by organ system and species. The percentage of positive samples in each organ system is shown below the name of that system in the leftmost column. Numbers not in parentheses refer to how many samples of the specific tissue from the indicated cetacean were analyzed, and numbers in parentheses refer to how many HV sequences were detected. Thus, “7 (2G)” means that two sequences were identified in seven analyzed samples, and that both belonged to *Gammaherpesvirinae*. The notation “2G1” means that the sequence G1 was detected twice in the given tissue and species. Sc, *Stenellacoeruleoalba*; Tt, *Tursiops truncatus*; Gg, *Grampus griseus*; Zc, *Ziphius cavirostris*. The bold parts refer to the total of individuals studied, tissues analyzed and types of sequences detected.

			Striped Dolphin						Bottlenose Dolphin		Risso’s Dolphin		Cuvier’s Beaked Whale	Total
			35 Sc						8 Tt		3 Gg		1 Zc	47 Individuals
Tissue			694 Tissues						149 Tissues		100 Tissues		23 Tissues	966 Tissues
Reproductive system (39.47%)	Mammary gland		1						2 (1G)		-		-	**3** **(1G)**
									1G1					
	Uterus		-						-		-		1 (1A)	**1** **(1A)**
													1A8	
	Ovary		3 (1G)						-		1 (1G)		-	**4** **(2G)**
			1G1								1G1			
	Testicle		4						1		-		-	**5**
	Penis		7 (3G)						3 (1G)		2 (1A)		-	**12** **(1A, 4G)**
			3G1						1G1		1A4			
	External genital mucosa		7 (2G)						2 (2G)		2 (1G)		1 (1A)	**12** **(1A, 5G)**
			2G1						2G1		1G1		1A8	
	External genital secretion		-						-		1		-	**1**
Nervous System (18.93%)	Optic nerve		4						-		-		-	**4**
	Meninges		2						4 (1A)		1		-	**7** **(1A)**
									1A2					
	Cerebral cortex		1						-		1		1 (1A)	**3** **(1A)**
													1A8	
	Brain (unspecified location)		34 (6G)						7 (2G)		2 (1A)		-	**43** **(1A, 8G)**
			5G1			1G2			2G1		1A9			
	Lateral ventricle		12 (1G)						2 (1G)		1		1 (1A)	**16** **(1A, 2G)**
			1G1						1G1				1A8	
	Thalamus		14 (2G)						2		3		1 (1A)	**20** **(1A, 2G)**
			2G1										1A8	
	Pons		17 (2A, 1G)						2 (1G)		3		1 (1A)	**23** **(3A, 2G)**
			1A1		1A10		1G1		1G1				1A8	
	Cerebellum		23 (1A, 3G)						5 (1G)		3 (1A)		1 (1A)	**32** **(3A, 4G)**
			1A1			3G1			1G1		1A9		1A8	
	Spinal cord		15 (2G)						2		3		1 (1A)	**21** **(1A, 2G)**
			2G1										1A8	
Tegument (14.40%)	Epidermis		62 (4A, 5G)						14 (1A, 3G)		6		1 (1A)	**83** **(6A, 8G)**
			1A1	2A3		1A4		5G1	1A5	3G1			1A11	
	Hipodermis		31 (1A, 2G)						7		3		1 (1A)	**42** **(2A, 2G)**
			1A1			2G1							1A11	
Endocrine system (13.95%)	Thyroid		9 (1G)						1		2		-	**12** **(1G)**
			1G1											
	Adrenal gland		23 (2A, 3G)						4		3		1	**31** **(2A, 3G)**
			1A1		1A2		3G1							
Circulatory system (12.28%)	Myocardium		13 (2G)						2		3 (1G)		1 (1A)	**19** **(1A, 3G)**
			2G1								1G1		1A8	
	Heart valve		2						2		1		-	**5**
	Pericardial sac		1						-		-		-	**1**
	Pericardial fluid		1						2		-		-	**3**
	Blood		12 (1A, 1G)						7 (1A)		3		-	**22** **(2A, 1G)**
			1A7			1G1			1A6					
	Serum		4						1		2		-	**7**
Urinary apparatus (11.63%)	Kidney		30 (1A)						7		4		1 (1A)	**42** **(2A)**
			1A1										1A8	
	Urinary bladder		25 (5G)						5		3 (1G)		1 (1A)	**34** **(1A, 6G)**
			5G1								1G1		1A8	
	Urine		2						4		3 (1G)		1	**10** **(1G)**
											1G1			
Digestive system (10.13%)	Oral cavity	Teeth and gingiva	30(4G)						7(1A)		2		-	**39** **(1A, 4G)**
			4G1						1A2					
		Tongue	16						-		3 (1G)		-	**19** **(1G)**
											1G1			
		Oral swab	3						-		2		-	**5**
	Digestive tube	Esophagus	-						-		2 (2G)		-	**2** **(2G)**
											2G2			
		Intestine	7						1		1		-	**9**
	Liver		31 (2G)						7 (2G)		3		1	**42** **(4G)**
			2G1						2G1					
	Bile		1						-		-		-	**1**
	Pancreas		24 (2G)						3		3		1 (1A)	**31** **(1A, 2G)**
			2G1										1A8	
Respiratory system (6.78%)	Blowhole swab		2						-		2		-	**4**
	Lung		39 (1A, 2G)						8		5		1 (1A)	**53** **(2A, 2G)**
			1A1			2G1							1A8	
	Lung exudate		2						-		-		-	**2**
Lymphatic system (4.64%)	Spleen		25						4		3		1	**33**
	Thymus		19 (1A)						4		1		-	**24** **(1A)**
			1A5											
	Pharyngeal tonsils		21 (1A, 2G)						2		4 (2G)		-	**27** **(1A, 4G)**
			1A1			2G1					1G1	1G3		
	Lymph nodes	Prescapular	26 (1A, 1G)						6		3		1 (1A)	**36** **(2A, 1G)**
			1A1			1G1							1A8	
		Pulmonar	32						6		3		1 (1A)	**42** **(1A)**
													1A8	
		Mesenteric	23 (1G)						5 (1A)		3		1	**32** **(1A, 1G)**
			1G1						1A1					
Musculoskeletal system (4.54%)	Epaxial muscle, thoracic region		32						8 (1G)		3		1 (1A)	**44** **(1A, 1G)**
									1G1				1A8	
Sense organs (0%)	Eye		2						-		1		-	**3**
TOTAL	**Positive tissues**		**70 of 694** **(16A 54G)** **10.09%**						**20 of 149** **(5A 15G)** **13.42%**		**13 of 100** **(3A 10G)** **13.00%**		**18 of 23** **(18A)** **78.26%**	**121 of 966** **(42A, 79G)** **12.53%**
	**Positive individuals**		**28 of 35** **80.00%**						**7 of 8** **87.50%**		**2 of 3** **66.67%**		**1 of 1** **100%**	**38 of 47** **80.85%**

**Table 2:** Relevant histopathological findings in samples with HV infection. Sc: *Stenellacoeruleoalba*, Tt: *Tursiopstruncatus*, Gg: *Grampusgriseus*, Zc: *Ziphiuscavirostris*, M: male, F: female, U: undetermined, N: neonate, C: calf, J: juvenile, A: adult, CNS: central nervous system, GHV: *Gammaherpesvirinae*, AHV: *Alphaherpesvirinae*, -: negative result in the determination, +: positive result in the determination.

Stranding Date	Species	Sex	Age	Sample	HVSF	HV Seq	RNA	Concomitant Lesions	Possible Differential Diagnosis
25.06.10	Tt	M	A	Liver	GHV	G1	-	Suppurative hepatitis	Bacterial
14.07.10	Tt	F	A	Urinary bladder	GHV	G1	-	None	-
				CNS	GHV	G1	+	None	-
				Mammary gland	GHV	G1	+	None	-
				Vagina	GHV	G1	+	Lymphoplasmacytic and hiperplastic vaginitis	Cetacean papillomavirus
				Skin	GHV	G1	+	Hyperplastic dermatitis	Cetacean poxvirus
				Skeletal muscle	GHV	G1	-	None	-
				Lymph nodes	GHV	G1	+	None	-
				Adrenal gland	GHV	G1	+	None	-
14.08.10	Sc	U	J	Skin	GHV	G1	+	Hyperplastic dermatitis with parasitic granuloma (nematodes)	-
12.03.11	Sc	M	N	Myocardium	AHV	A7	+	Degeneration	-
13.03.11	Sc	M	N	Lung	GHV	G1	-	Chronic interstitial pneumonia	CeMV
15.03.11	Sc	F	J	Urinary bladder	GHV	G1	-	Hemorrhagic cystitis	-
				Skin	GHV	G1	+	None	-
				Adrenal glands	GHV	G1	+	Bilateral lymphoplasmacytic adrenalitis	-
16.03.11	Sc	F	A	Urinary bladder	GHV	G1	+	Lymphoplasmacytic cystitis with intracytoplasmic inclusion bodies	CeMV
23.03.11	Sc	F	A	CNS	GHV	G1	+	Lymphoplasmacytic meningoencephalitis	CeMV, *Brucella ceti*, *Toxoplasma gondii*
23.03.11	Tt	M	J	CNS	AHV	A2	+	Lymphoplasmacytic meningitis	CeMV, *Brucella ceti*
				Skeletal muscle	GHV	G1	-	None	-
25.03.11	Sc	F	J	CNS	GHV	G1	+	Lymphoplasmacytic meningoencephalitis	CeMV, *Brucella ceti*, *Toxoplasma gondii*
				Skin	GHV	G1	+	Degenerative and necrotizing dermatitis	-
				Adrenal gland	AHV	A2	-	None	-
26.03.11	Sc	F	J	CNS	GHV	G1	+	Lymphoplasmacytic meningoencephalitis	CeMV, *Brucella ceti*, *Toxoplasma gondii*
				Skin	GHV	G1	-	Lymphoplasmacytic dermatitis	-
29.03.11	Sc	M	C	CNS	GHV	G1	-	Lymphoplasmacytic meningoencephalitis	-
18.06.11	Sc	M	J	Skin	AHV	A3	+	Hyperplastic dermatitis	Cetacean poxvirus
07.07.11	Sc	M	J	CNS	GHV	G1	+	Lymphoplasmacytic meningoencephalitis	CeMV, *Brucella ceti*, *Toxoplasma gondii*
				Skin	GHV	G1	+	Hyperplastic dermatitis	-
29.07.11	Sc	F	A	Pancreas	GHV	G1	-	None	-
				Liver	GHV	G1	-	Chronic cholangiohepatitis	Trematode
12.10.11	Sc	M	J	Urinary bladder	GHV	G1	+	Lymphoplasmacytic cystitis	-
				Vaginal mucosa	GHV	G1	+	Lymphoplasmacytic vaginitis	-
11.12.11	Gg	M	A	Penis	AHV	A4	-	Proliferative balanitis	Cetacean papillomavirus
				Esophagus	GHV	G2	-	Proliferative esophagitis	Cetacean papillomavirus
19.12.11	Sc	F	C	CNS	GHV	G2	-	Lymphoplasmacytic meningitis	CeMV, *Brucella ceti*
26.01.12	Sc	M	C	Kidney	AHV	A1	-	Lymphoplasmacytic nephritis	-
				CNS	AHV	A1	+	None	-
				Lung	AHV	A1	-	Interstitial bronchopneumonia with associated granulomatous bronchopneumonia	CeMV, Nematodes
				Lymph node	AHV	A1	-	Granulomatous lymphadenitis	Parasitic migration
22.03.12	Sc	F	J	CNS	GHV	G1	-	Lymphoplasmacytic meningoencephalitis	CeMV, *Brucella ceti*, *Toxoplasma gondii*
				Myocardium	GHV	G1	-	None	-
15.05.12	Zc	F	J	Kidney	AHV	A8	-	Interstitial nephritis	-
				CNS	AHV	A8	+	None	-
				Lung	AHV	A8	-	Fibrinonecrotic bronchopneumonia	Aspiration of fibrinonecrotic content
				Uterus	AHV	A8	-	None	-
				Skin	AHV	A11	-	None	-
				Lymph node	AHV	A8	-	Lymphoid depletion	-
				Myocardium	AHV	A8	-	None	-
07.09.12	Sc	M	N	Liver	GHV	G1	-	None	-
20.10.12	Sc	M	J	Myocardium	GHV	G1	-	None	-
13.02.13	Sc	M	A	Skin	AHV	A4	+	Hyperplastic dermatitis	Cetacean poxvirus
29.03.13	Gg	F	J	Urinary bladder	GHV	G1	+	None	-
				CNS	AHV	A9	-	None	-
				Myocardium	GHV	G1	-	None	-
12.05.13	Sc	M	J	Penis	GHV	G1	+	None	-

## Data Availability

DNA sequences have been deposited in GenBank (accession numbers: KP995680, KP995681, KP995682, KP995683, KP995684, and KP995685).
